# Incidence and Characteristics in Children with Post–COVID-19 Condition in Sweden

**DOI:** 10.1001/jamanetworkopen.2023.24246

**Published:** 2023-07-19

**Authors:** Maria Bygdell, Jenny M. Kindblom, Jari Martikainen, Huiqi Li, Fredrik Nyberg

**Affiliations:** 1 Department of Internal Medicine and Clinical Nutrition, Institute of Medicine, Sahlgrenska Academy, University of Gothenburg, Gothenburg, Sweden; 2 School of Public Health and Community Medicine, Institute of Medicine, Sahlgrenska Academy, University of Gothenburg, Gothenburg, Sweden; 3 Department of Drug Treatment, Sahlgrenska University Hospital, Region Västra Götaland, Gothenburg, Sweden; 4 Bioinformatics and Data Centre, Sahlgrenska Academy, University of Gothenburg, Gothenburg, Sweden

## Abstract

This cohort study of health data from 2 regions in Sweden examines incidence rates of post–COVID-19 among children and compares incidence rates by demographic and clinical characteristics.

## Introduction

During the COVID-19 pandemic it has become increasingly clear that children have not been as severely affected as adults in the acute phase of the infection, with the exception of those affected by multisystem inflammatory syndrome in children (MIS-C).^1,2^ In accordance with adults, some children seem to be affected by persistent long-term symptoms of COVID-19, commonly referred to as post–COVID-19 condition (PCC).^3^ The frequency of PCC in the pediatric population is to a large extent unknown, and the occurrence in subgroups is not completely understood. The aim of this descriptive study was to describe the incidence of PCC in children and different subgroups of children using a unique population-based cohort with near-complete follow-up in high-quality Swedish registers.

## Methods

We included all children ages 6 to 17 years residing in the 2 largest Swedish regions and retrieved information on both inpatient and outpatient care from specialists and primary health care clinicians using high-quality national or regional registers with near-complete coverage (eMethods in Supplement 1). The inclusion criterion was COVID-19 infection between January 31, 2020, and February 9, 2022. PCC was defined by *International Statistical Classification of Diseases and Related Health Problems, Tenth Revision *(ICD-10) code U09.9 (used in Sweden since October 2020^4,5^) as the main or secondary diagnosis occurring 28 days or more after the COVID-19 infection. Follow-up ended at the earliest of: PCC diagnosis, emigration, death, or end of study (November 30, 2022). Incidence rate (per 100 person-years) and cumulative incidence are presented for the total cohort and according to subgroups. Data were analyzed with R Statistical Software version 4.2.2 (R Project for Statistical Computing).

## Results

A total of 162 383 children (81 789 boys [50.4%], 80 594 girls [49.6%]), with a mean (SD) age of 12.0 (3.5) years at study start, had experienced a COVID-19 infection. Only a small proportion (529 [0.3%]) had been hospitalized due to COVID-19 and none of the children with PCC had been treated in intensive care. We found a PCC diagnosis in 326 children (0.2%) with COVID-19 (Figure). We observed numerically higher incidence rates of PCC cases among girls than boys (incidence rate per 100 person-years: 0.19; 95% CI, 0.16-0.22 vs 0.12; 95% CI, 0.10-0.14), older than younger children (age 12 to 17 years, 0.19; 95% CI, 0.17-0.22 vs 0.11; 95% CI, 0.09-0.14), with vs without comorbidities (any, 0.16; 95% CI, 0.14-0.18 vs none, 0.11; 95% CI, 0.07-0.15), and among hospitalized compared with nonhospitalized for acute COVID-19 (1.25; 95% CI, 0.62-2.23 vs 0.15; 95% CI, 0.13-0.17) (Table). Similar PCC occurrence was seen across categories of parental education. Children who had a diagnosis of MIS-C (63 children) showed an increased occurrence of PCC diagnosis compared with children without MIS-C. In addition, we observed a 6-fold higher occurrence of PCC if any of the parents had a PCC diagnosis (Table).

**Figure.**
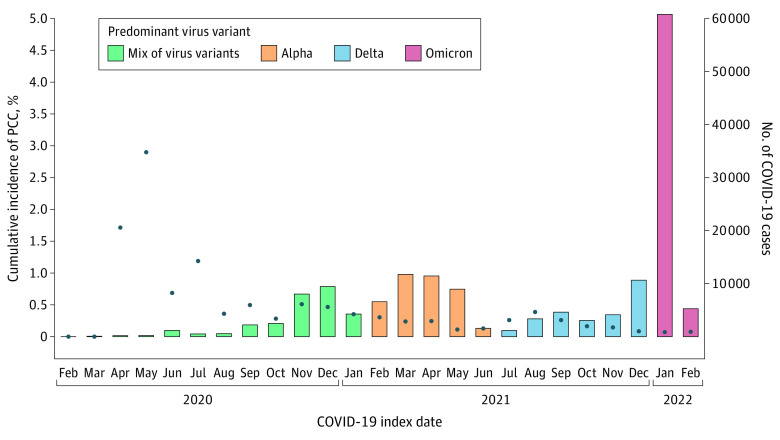
Total COVID-19 Cases and Incidence of of Post–COVID-19 Condition (PCC) by Date Number of incident cases of COVID-19 (bars) and proportion of COVID-19 cases developing PCC (blue dots) among children ages 6 to 17 years with COVID-19 from the 2 largest regions in Sweden, Stockholm and Västra Götaland, based on both primary health care data and inpatient and outpatient specialist health care data, until November 30, 2022. Specific time periods of predominant virus variants of concern indicated. Full-scale polymerase chain reaction testing was not implemented in Sweden until late August 2020, therefore, the percentage of PCC cases during the first 7 months should be interpreted with caution.

**Table.** Cohort Incidence Rate and Cumulative Incidence of PCC Diagnosis

Characteristics	During follow-up, No.		Occurrence of PCC	
	PCC	No PCC	Incidence rate per 100 person-years (95% CI)	Cumulative incidence, % (95% CI)
Overall	326	162 057	0.15 (0.14-0.17)	0.20 (0.18-0.22)
Sex				
Boys	128 (39.2)	81 661 (50.4)	0.12 (0.10-0.14)	0.16 (0.13-0.19)
Girls	198 (60.7)	80 396 (49.6)	0.19 (0.16-0.22)	0.25 (0.21-0.28)
Age group^a^				
6-11 y	113 (34.7)	83 495 (51.5)	0.11 (0.09-0.14)	0.14 (0.11-0.16)
12-17 y	211 (64.7)	78 564 (48.4)	0.19 (0.17-0.22)	0.27 (0.23-0.31)
Comorbidities				
Any^b^	300 (92.0)	142 894 (88.2)	0.16 (0.14-0.18)	0.21 (0.19-0.24)
None	26 (8.0)	19 163 (11.8)	0.11 (0.07-0.15)	0.14 (0.09-0.20)
Asthma and allergy^c^	50 (15.3)	17 885 (11.0)	0.21 (0.16-0.28)	0.28 (0.21-0.37)
No asthma and allergy	276 (84.7)	144 172 (89.0)	0.15 (0.13-0.17)	0.19 (0.17-0.22)
Psychiatric conditions^d^	58 (17.8)	20 667 (12.8)	0.21 (0.16-0.27)	0.28 (0.21-0.36)
No psychiatric conditions	268 (82.2)	141 390 (87.2)	0.15 (0.13-0.16)	0.19 (0.17-0.21)
Diabetes^e^	2 (0.6)	762 (0.5)	0.19 (0.02-0.68)	0.26 (0.05-1.05)
No diabetes	324 (99.4)	161 295 (99.5)	0.15 (0.14-0.17)	0.20 (0.18-0.22)
Acute COVID-19 care level				
Hospitalized^f^	11 (3.4)	518 (0.3)	1.25 (0.62-2.23)	2.08 (1.10-3.80)
Nonhospitalized	315 (96.6)	161 539 (99.7)	0.15 (0.13-0.17)	0.20 (0.17-0.22)
MIS-C^g^	6 (1.8)	57 (<0.1)	5.81 (2.13-12.64)	9.52 (3.93-20.24)
No MIS-C	320 (98.2)	162 000 (>99.9)	0.15 (0.14-0.17)	0.20 (0.18-0.22)
Virus variant^h^				
Mix of virus variants	143 (43.9)	28 982 (17.9)	0.24 (0.20-0.28)	0.49 (0.42-0.58)
Alpha	88 (27.0)	40 192 (24.8)	0.13 (0.11-0.16)	0.22 (0.18-0.27)
Delta	48 (14.7)	26 894 (16.6)	0.16 (0.12-0.22)	0.18 (0.13-0.24)
Omicron	47 (14.4)	65 989 (40.7)	0.08 (0.06-0.11)	0.07 (0.05-0.10)
Parents’ maximum education				
Primary school	8 (2.5)	6077 (3.7)	0.10 (0.04-0.19)	0.13 (0.06-0.27)
Secondary school	84 (25.8)	38 260 (23.6)	0.16 (0.13-0.20)	0.22 (0.18-0.27)
Tertiary school	165 (50.6)	81 962 (50.6)	0.15 (0.13-0.18)	0.20 (0.17-0.24)
Unknown	69 (21.2)	35 758 (22.1)	0.15 (0.12-0.20)	0.19 (0.15-0.25)
Any parent diagnosed with PCC				
Yes	35 (10.7)	3401 (2.1)	0.70 (0.49-0.97)	1.02 (0.72-1.43)
No	291 (89.3)	158 656 (97.9)	0.14 (0.13-0.16)	0.18 (0.16-0.21)

Abbreviations: MIS-C, multisystem inflammatory syndrome in children; PCC, post–COVID-19 condition.

^a^ At study start (January 31, 2020).

^b^ Defined as any diagnosis *International Statistical Classification of Diseases and Related Health Problems, Tenth Revision *(*ICD-10*) code during 2018-2019.

^c^ Defined as *ICD-10* code J30 or J45 during 2018-2019.

^d^ Defined as *ICD-10* code F00-F99 during 2018-2019.

^e^ Defined as *ICD-10* code E10-E14 during 2018-2019.

^f^ Including both hospitalization at intensive care units (0 and 40) and regular wards (11 and 478).

^g^ Defined as *ICD-10* code U10.9. A total of 6 children received both a PCC diagnosis and MIS-C diagnosis, with 4 receiving a PCC diagnosis more than 28 days after MIS-C diagnosis.

^h^ Dominant virus variant during the time period when the acute COVID-19 infection occurred.

## Discussion

In this population-based, well-powered cohort with uniquely comprehensive data regarding COVID-19 and PCC, only 0.2% children with COVID-19 had a subsequent diagnosis of PCC. Children with PCC were more often girls, older, had comorbidities, had been hospitalized for their COVID-19, or had a parent with a PCC diagnosis. The higher cumulative incidence of PCC among children with a parent with PCC may indicate that the etiology of PCC could involve genetic susceptibility, or that parental experience of long-term symptoms raises awareness of symptoms and facilitates navigation of the health care system. Moreover, the increased occurrence of PCC among hospitalized children is well in accordance with similar results regarding hospitalized adults.^6^ Because none of the children with PCC had been treated in intensive care, the diagnosis cannot be explained by misclassification of post–intensive care syndrome. The number of MIS-C cases in the study population was low and therefore the association of MIS-C with PCC needs to be evaluated further. A limitation with our study was that the PCC diagnosis code is not yet validated, and the strengths include the unique comprehensive data, the large population-based cohort, and the near-complete follow-up in high-quality registers.

In this descriptive study, PCC in children was rare. We observed a higher number of children with female sex, older age, hospitalization for COVID-19, and having a parent with PCC among cases with PCC.

## Notes

Supplement 1.
eMethods.

Supplement 2.
Data Sharing Statement

## References

[1] Viner RM, Mytton OT, Bonell C, . Susceptibility to SARS-CoV-2 infection among children and adolescents compared with adults: a systematic review and meta-analysis. JAMA Pediatr. 2021;175(2):143-156. doi:10.1001/jamapediatrics.2020.4573 32975552 PMC7519436

[2] Abrams JY, Oster ME, Godfred-Cato SE, . Factors linked to severe outcomes in multisystem inflammatory syndrome in children (MIS-C) in the USA: a retrospective surveillance study. Lancet Child Adolesc Health. 2021;5(5):323-331. doi:10.1016/S2352-4642(21)00050-X 33711293 PMC7943393

[3] World Health Organization. Emergency use ICD codes for COVID-19 disease outbreak. Accessed June 28, 2022. https://www.who.int/standards/classifications/classification-of-diseases/emergency-use-icd-codes-for-covid-19-disease-outbreak

[4] World Health Organization. Updates 3 & 4 in relation to COVID-19 coding in ICD-10. Accessed June 28, 2022. https://cdn.who.int/media/docs/default-source/classification/icd/covid-19/covid-19-coding-updates-3-4-combined.pdf?sfvrsn=39197c91_3

[5] National Board of Health and Welfare. Statistik om tillstånd efter COVID-19. 2021. Article in Swedish. April 15, 2021. Accessed June 28, 2022. https://www.socialstyrelsen.se/globalassets/sharepoint-dokument/artikelkatalog/ovrigt/2021-4-7353.pdf

[6] Bygdell M, Leach S, Lundberg L, . A comprehensive characterization of patients diagnosed with post–COVID-19 condition in Sweden 16 months after the introduction of the *International Classification of Diseases Tenth Revision* diagnosis code (U09.9): a population-based cohort study. Int J Infect Dis. 2023;126:104-113. doi:10.1016/j.ijid.2022.11.021 36410693 PMC9678230

