# Supplementary material for: Incidence and Characteristics in Children with Post–COVID-19 Condition in Sweden
Source: JAMA Netw Open. 2023 Jul 19;6(7):e2324246. doi: 10.1001/jamanetworkopen.2023.24246 (PMC10357335; doi:10.1001/jamanetworkopen.2023.24246)
Supplement: Supplement 1. — eMethods. [file jamanetwopen-e2324246-s001.pdf]

Supplemental Online Content

Bygdell M, Kindblom JM, Martikainen J, Li H, Nyberg F. Incidence and characteristics in children with post–COVID-19 condition in Sweden. *JAMA Netw Open.* 2023;6(7):e2324246. doi:10.1001/jamanetworkopen.2023.24246

eMethods.

This supplemental material has been provided by the authors to give readers additional information about their work.

© 2023 Bygdell M et al. *JAMA Network Open.*

eMethods

The present study is part of the SCIFI-PEARL (Swedish Covid-19 Investigation for Future Insights – a Population Epidemiology Approach using Register Linkage) project, described in more detail elsewhere[1]. At birth or at immigration, every citizen in Sweden is assigned a unique personal identity number (PIN) that follows the individual throughout life. Using the PIN, data from many registers are linked in the SCIFI-PEARL project. The registers from which data are included in the present study are from the National Board of Health and Welfare (National Patient Register [NPR] that includes all inpatient and specialist outpatient healthcare in Sweden)[2], Statistics Sweden (Longitudinal integrated database for health insurance and labour market studies [LISA] with sociodemographic and socioeconomic data[3], Multigenerational Register with data on parent/child connections, Register of the total population for age, sex and basic demographics)[4], the Public Health Agency (National Register of Notifiable Diseases [SmiNet] a register of all notifiable communicable diseases including all positive SARS-CoV-2 polymerase chain reaction [PCR] test results)[5]. In addition, data from the Swedish Intensive Care Register (SIR) and the regional primary healthcare databases in Stockholm (VAL database) and Västra Götaland (VEGA database) are included. The present register-based study was approved by the Swedish Ethical Review Authority who waived the requirement of written informed consent. We followed the STROBE guidelines for reporting of cohort studies where applicable.

A COVID-19 infection was defined as having a positive SARS-CoV-2 PCR test result registered in SmiNet and/or having the International Classification of Diseases, Tenth Revision (ICD-10) diagnosis COVID-19 (U07.1 or U07.2, as main or secondary diagnosis) in NPR/VEGA/VAL during the study inclusion period 31 January 2020 until 9 February 2022 (end of full population PCR testing in Sweden). The first registration of any of these was defined as the COVID-19 index date. We required a minimum follow-up of 220 days (corresponding to the third quartile of follow-up between COVID-19 index date and PCC index date among PCC patients diagnosed after the introduction of the PCC diagnosis code) from COVID-19 index date until death, emigration, or end of the study (n=239 not fulfilling this requirement).

In the acute phase of COVID-19, severity was categorised as either hospitalised (requiring intensive care or requiring non-intensive inpatient care), or not hospitalised (requiring neither intensive nor inpatient care). This categorisation was based on data from SIR and NPR's inpatient register. Sociodemographic data such as age, residency, sex, and parents' education were obtained from the LISA database (including the Register of the Total Population). Age was defined from birth until the start of the study, 31 January 2020, and categorised into two groups (6-11 and 12-17 years of age). Education was categorised into primary school level (<10 years), secondary school level (10-12 years), tertiary school level (>12 years), and unknown. Data on comorbidities were retrieved from NPR from 1 January 2018 until 31 December 2019. First, we divided the study population into two groups, having a diagnosis during 2018-2019 or not (any comorbidity vs. none). Then, we divided the study population into having a diagnosis during 2018-2019 or not, according to the most common diagnoses among children in Sweden (asthma and allergy [ICD-codes: J30 or J45], psychiatric disease [ICD-codes: F00-F99], and diabetes [ICD-codes: E10-E14]).

© 2023 Bygdell M et al. *JAMA Network Open.*

Each patient's COVID-19 index date was categorized according to distinct time periods corresponding to the dominant virus variant. In Sweden, a combination of virus variants predominated from February 2020 to January 2021, followed by the Alpha variant of concern (VOC) from February 2021 to June 2021, the Delta VOC from July 2021 to December 2021, and the Omicron VOC from January 2022 until end of inclusion[6].

References

1. Nyberg F, Franzen S, Lindh M, et al. Swedish Covid-19 Investigation for Future Insights - A Population Epidemiology Approach Using Register Linkage (SCIFI-PEARL). *Clin Epidemiol*. 2021;13:649-659. doi:10.2147/CLEP.S312742
2. Ludvigsson JF, Andersson E, Ekbom A, et al. External review and validation of the Swedish national inpatient register. *BMC Public Health*. Jun 9 2011;11(1):450. doi:10.1186/1471-2458-11-450
3. Ludvigsson JF, Svedberg P, Olen O, Bruze G, Neovius M. The longitudinal integrated database for health insurance and labour market studies (LISA) and its use in medical research. *Eur J Epidemiol*. Apr 2019;34(4):423-437. doi:10.1007/s10654-019-00511-8
4. Ludvigsson JF, Almqvist C, Bonamy AK, et al. Registers of the Swedish total population and their use in medical research. *Eur J Epidemiol*. Feb 2016;31(2):125-36. doi:10.1007/s10654-016-0117-y
5. Rolfhamre P, Jansson A, Arneborn M, Ekdahl K. SmiNet-2: Description of an internet-based surveillance system for communicable diseases in Sweden. *Euro Surveill*. 2006;11(5):103-7.
6. Public Health Agency. Statistik om SARS-CoV-2 virusvarianter av särskild betydelse. https://www.folkhalsomyndigheten.se/smittskydd-beredskap/utbrott/aktuella-utbrott/covid-19/statistik-och-analyser/sars-cov-2-virusvarianter-av-sarskild-betydelse/

© 2023 Bygdell M et al. *JAMA Network Open.*
