# Supplementary material for: Incidence and Characteristics in Children with Post–COVID-19 Condition in Sweden
Source: JAMA Netw Open. 2023 Jul 19;6(7):e2324246. doi: 10.1001/jamanetworkopen.2023.24246 (PMC10357335; doi:10.1001/jamanetworkopen.2023.24246)
Supplement: Supplement 2. — Data Sharing Statement [file jamanetwopen-e2324246-s002.pdf]

Data Sharing Statement

Bygdell. Incidence and Characteristics in Children with Post-COVID-19 Condition in Sweden. *JAMA Netw Open*. Published July 19, 2023. doi:10.1001/jamanetworkopen.2023.24246

Data
Data available: No

Additional Information
Explanation for why data not available: The data used in this study are deidentified individual-level data from Swedish healthcare registers and can be obtained from the respective Swedish public data holders on the basis of ethics approval for the research in question, subject to relevant legislation, processes, and data protection.
